# Amarogentin Displays Immunomodulatory Effects in Human Mast Cells and Keratinocytes

**DOI:** 10.1155/2015/630128

**Published:** 2015-10-27

**Authors:** Ute Wölfle, Birgit Haarhaus, Christoph M. Schempp

**Affiliations:** Research Center skinitial, Department of Dermatology, University Medical Center, 79104 Freiburg, Germany

## Abstract

Keratinocytes express the bitter taste receptors TAS2R1 and TAS2R38. Amarogentin as an agonist for TAS2R1 and other TAS2Rs promotes keratinocyte differentiation. Similarly, mast cells are known to express bitter taste receptors. The aim of this study was to assess whether bitter compounds display immunomodulatory effects on these immunocompetent cells in the skin, so that they might be a target in chronic inflammatory diseases such as atopic dermatitis and psoriasis. Here, we investigated the impact of amarogentin on substance P-induced release of histamine and TNF-*α* from the human mast cell line LAD-2. Furthermore, the effect of amarogentin on HaCaT keratinocytes costimulated with TNF-*α* and histamine was investigated. Amarogentin inhibited in LAD-2 cells substance P-induced production of newly synthesized TNF-*α*, but the degranulation and release of stored histamine were not affected. In HaCaT keratinocytes histamine and TNF-*α* induced IL-8 and MMP-1 expression was reduced by amarogentin to a similar extent as with azelastine. In conclusion amarogentin displays immunomodulatory effects in the skin by interacting with mast cells and keratinocytes.

## 1. Introduction

Mast cells are strategically located in the upper dermis of normal skin, where host tissue is exposed to external antigens and bacteria. After activation by a range of stimuli (e.g., cross-linking of the IgE receptors and binding of the neuropeptide substance P released from sensory nerve fibers in the skin during inflammation) mast cells release mediators such as histamine [1]. Histamine is also stored in large amounts in secretory glands. It is involved in the elicitation of immediate-type allergic reactions as well as in tissue remodeling and chronic inflammation [2, 3] by binding to one of the four known G-protein coupled transmembrane H1–H4 receptors. These receptors are expressed on various cell types including monocytes, lymphocytes, dendritic cells, and keratinocytes [4]. Furthermore, mast cells can synthesize de novo a range of cytokines such as TNF-*α*, growth factors, and membrane molecules involved in inflammatory reactions [2]. In healthy skin only a few mast cells are present in the upper dermis. However, the number of mast cells as well as the histamine level increases in chronic skin inflammation such as psoriasis, chronic leg ulcers, and epithelial skin cancer [2]. Besides the involvement in immediate-type allergy and maturation of dendritic cells, histamine also stimulates human keratinocytes through the H1 receptor to increase the expression of the proinflammatory cytokine IL-6, the chemokine IL-8 [5], the nerve growth factor (NGF) [6], and matrix metalloproteinases (MMPs), especially MMP-1 and MMP-9 [7, 8]. MMP-1, the so-called interstitial collagenase, specifically cleaves type 1 collagen, a major constituent of the dermis. MMP-1 also activates other MMPs such as MMP-9 that has the highest substrate specificity for dermal elastin and fibrillin [9]. The cleavage of the components in the basement membrane allows T cells to cross the basement membrane and enter the epidermal compartment during skin inflammation. Mast cells, T cells, and keratinocytes interact with one another in the uppermost dermis of inflamed skin. In the epidermis of lesional skin from patients with atopic dermatitis cutaneous nerve fibres are present at high densities. Their length is increased and they reach the surface of the skin [10]. This abnormal innervation is thought to be one cause of the intense itching of atopic skin. One of the mediators involved in nerve fibre expansion is NGF that is released from epidermal keratinocytes and accumulates in the epidermis. Strong expression of NGF and its receptors is also observed throughout the entire epidermis on a psoriatic lesion. Under physiological conditions keratinocyte-derived NGF plays an important role in the maintenance and regeneration of cutaneous nerves in normal skin, normal proliferation and differentiation of cutaneous nerves, keratinocytes, and melanocytes [11]. However, NGF production in keratinocytes can be increased by histamine and TNF-*α* released from mast cells [6, 11]. Kumar and colleagues reported that amarogentin, a secoiridoid glycoside that is present in the Indian plant* Swertia chirayita,* modulates in arthritic mice the secretion of proinflammatory cytokines including TNF-*α* [12]. However, it is unclear whether amarogentin the bitterest substance in nature that is also present in high amount in the alpine flora* Gentiana lutea* can also modulate immune reactions in inflamed skin. As amarogentin is an agonist for several bitter taste receptors (TAS2R1, TAS2R4, TAS2R39, TAS2R43, TAS2R46, TAS2R47, and TAS2R50) [13] and the expression of at least the bitter taste receptors TAS2R1 and TAS2R38 can be found on keratinocytes, amarogentin could influence cutaneous inflammation. Recently we showed already that amarogentin enhances keratinocyte differentiation [14]. In this study we analyzed if amarogentin might also influence the release of histamine and TNF-*α* by mast cells and/or the interaction of these proinflammatory stimuli with keratinocytes. In this way amarogentin could be a target in chronic inflammatory diseases such as atopic dermatitis and psoriasis.

## 2. Material and Methods

### 2.1. Cytotoxicity Test

Cytotoxicity of amarogentin and azelastine in LAD-2 cells was assessed with the ViaLight Plus ATP assay (Cambrex, Verviers, Belgium) according to the manufacturer's instructions. The method is based on the bioluminescence measurement of ATP that is present in metabolically active cells. Luciferase catalyzes the formation of light from ATP and luciferin. The emitted light intensity is directly proportional to the ATP concentration and is measured using a luminometer (Sirius HT; MWG).

### 2.2. Cell Culture

LAD-2 human mast cells (kindly provided by Dr. A. Kirshenbaum, National Institute of Allergy and Infectious Diseases, Bethesda, MD, USA) were cultured in serum-free media (StemPro-34, Thermo Fisher Scientific, Darmstadt, Germany) supplemented with 2 mM L-glutamine and 100 ng/mL rhSCF (recombinant human stem cell factor; Cell Signaling Technologies). The human keratinocyte cell line HaCaT was cultured in Dulbecco's modified essential medium (DMEM; Invitrogen GmbH, Karlsruhe, Germany) containing 10% fetal calf serum (FCS; PAA, Pasching, Austria). All cells were cultivated at 37°C in a humidified atmosphere with 5% CO_2_.

### 2.3. Human Mast Cell Stimulation

LAD-2 cells were washed with PBS and resuspended in the appropriate medium. LAD-2 cells (2 × 10^5^ cells/400 *μ*L/well) were plated in 48-well flat bottom Falcon cell culture plates from Becton Dickinson and then stimulated with SP (2 *μ*M, Sigma-Aldrich, Seelze, Germany) for 1 or 24 h. Some cells were preincubated for 30 min with amarogentin (100 *μ*M, Phytolab, Vestenbergsgreuth, Germany) or azelastine (24 *μ*M, Sigma-Aldrich) as indicated. A part of the amarogentin stimulated group was preincubated for 30 min with the PLC inhibitor U73122 (10 *μ*M, Sigma-Aldrich). The supernatants were collected for further assays.

### 2.4. Human Keratinocyte Stimulation

HaCaT cells (2 × 10^5^ cells/400 *μ*L/well) were plated in 48-well flat bottom Falcon cell culture plates from Becton Dickinson and then stimulated with histamine (10 *μ*M) and TNF-*α* (25 ng/mL) or IFN-*γ* (200 U/mL) for 24 h. Some cells were preincubated for 30 min with amarogentin or azelastine (24 *μ*M) as indicated. A part of the amarogentin stimulated group was preincubated for 30 min with the PLC inhibitor U73122 (10 *μ*M, Sigma-Aldrich). The supernatants were collected for further assays.

### 2.5. Degranulation Assays

Mast cell degranulation was assessed by measuring histamine in the supernatant fluid 1 h after cell stimulation with SP (2 *μ*M). Histamine levels were assayed 20 min after SP stimulation using a histamine ELISA from IBL international.

### 2.6. Cytokine and MMP-1 Release Assays

TNF-*α*, IL-6, IL-8, and MMP-1 release into the supernatant fluid 24 h after cell stimulation (either LAD-2 or HaCaT cells) was measured by Enzyme-Linked Immunosorbent Assay (ELISA) using a commercial kit from R&D Systems according to the manufacturer's instructions.

### 2.7. RNA Extraction and PCR

HaCaT cells were preincubated with amarogentin (100 mM) or azelastine (24 *μ*M) for 2 h at 37°C. Then the cells were stimulated with histamine (10 *μ*M) and TNF-*α* (25 ng/mL). After 3 h at 37°C total RNA was extracted with the RNeasy Mini kit (Quiagen, Hilden, Germany). First-strand cDNA was synthesized from 2 *μ*g total RNA in 20 *μ*L final volume using the Omniscript kit (Qiagen, Hilden, Germany) with random hexamer primers (Invitrogen). 2 *μ*L aliquots of the reverse transcription solution was used as a template for specific PCR reactions with an annealing temperature of 58°C, the PCR product was analyzed by gel electrophoresis, and the bands were quantified with image J. The PCR primers (20 pMol each) used to amplify NGF and the house keeping gene *β*-actin were

 NGF forward primer: 5′-aagcggcgactccgttcacc-3′, reverse primer: 5′-ggagcgtgtcggcaggtcag-3′;
 
*β*-actin forward primer: 5′-cgagcacagagcgtcgccttt-3′; reverse primer: 5′-gaccccgtcaccggagtcca-3′.

### 2.8. Immunohistochemistry

LAD-2 cells were fixed and permeabilized and immunostaining was performed with the polyclonal rabbit anti-human TAS2R1 antibody (Abcam, Cambridge, UK) at a concentration of 1 : 1000. Application of the primary antibody (4°C, overnight) was followed by incubation with biotinylated swine anti-goat, anti-mouse, and anti-rabbit antibody immunoglobulins (1 h, RT), streptavidin conjugated to horseradish peroxidase (20 min, RT), AEC solution as chromogen, and hematoxylin counterstaining. Staining with the rabbit immunoglobulin fraction served as isotype control. Images were taken with a microscope (Carl Zeiss AG, Oberkochen, Germany) equipped with Axiovision software.

### 2.9. Statistical Analysis

The data from all the procedures were expressed as the average ± standard error using Excel. Two group comparisons were evaluated using unpaired *t*-test. In all analyses, *p* ≤ 0.05 was considered statistically significant (*∗*); 0.05 < *p* < 0.06 was considered as borderline significant (bs) and *p* > 0.06 was considered as not statistically significant (ns).

## 3. Results

### 3.1. Effect of Amarogentin on Histamine and Cytokine Release from Mast Cells

**Figure 1:**
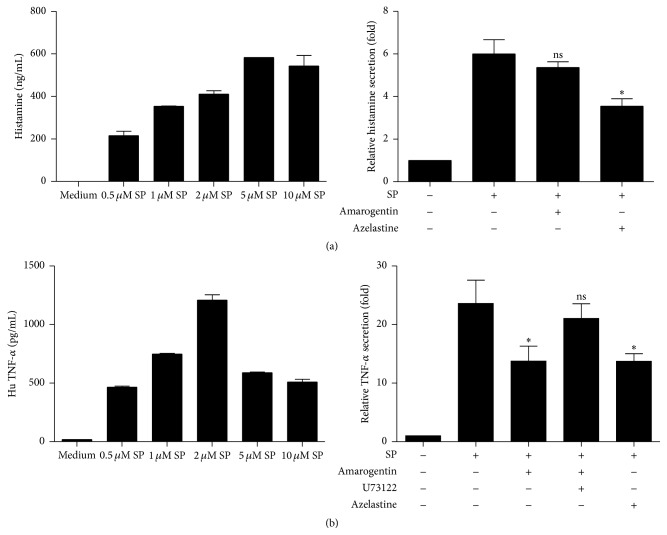
Amarogentin inhibits substance P- (SP-) triggered mediator release from the human mast cell line LAD-2. LAD-2 cells were stimulated with increasing concentrations of SP. LAD-2 cells stimulated with 2 *μ*M SP were preincubated with amarogentin (100 *μ*M) or the positive control azelastine (24 *μ*M). A part of the amarogentin stimulated group was preincubated for 30 min with the PLC inhibitor U73122 (10 *μ*M). Histamine (a) and TNF-*α* (b) release was measured 20 min after SP stimulation. The data are shown with standard deviation (SEM) and were analyzed with the unpaired, two-tailed *t*-test (^*∗*^ *p* < 0.05; ns: not statistically significant; *n* = 3).

Substance P (SP) augments the release of histamine from the human leukemic LAD-2 mast cell line 1 h after stimulation in a dose dependent manner. Furthermore, SP induced the production of TNF-*α* after 24 h (Figures 1(a) and 1(b), first graph). 2 *μ*M SP was the best concentration to induce histamine and TNF-*α* release and was used for all further experiments.

To test if the bitter compound amarogentin can inhibit histamine or TNF-*α* release, LAD-2 cells were incubated with 100 *μ*M amarogentin according to former experiments and the literature [13, 14]. As positive control, we applied the histamine-1 receptor antagonist azelastine at a concentration of 24 *μ*M referring to data from the literature [15]. Both concentrations of amarogentin and azelastine were not cytotoxic for LAD-2 cells (supplementary data 1 in Supplementary Material available online at http://dx.doi.org/10.1155/2015/630128). In contrast to azelastine (24 *μ*M, 30 min) preincubation with amarogentin (100 *μ*M, 30 min) did not inhibit histamine release (Figure 1(a)). However, preincubation with both amarogentin (100 *μ*M, 30 min) and azelastine (24 *μ*M, 30 min) blocked in LAD-2 mast cells the secretion of newly synthesized TNF-*α* 24 h after SP (2 *μ*M) stimulation (Figure 1(b)). This demonstrates that amarogentin does not inhibit the degranulation of mast cell stored mediators, but it inhibits the new synthesis of TNF-*α*. To test if this inhibitory effect of amarogentin is mediated via bitter taste receptor signaling we used U73122, a phospholipase C inhibitor that was already described as inhibitor of the bitter taste receptor signalling in rat neuronal PC12 cells [16]. According to the literature the used concentration of U73122 was 10 *μ*M. It could be shown that U73122 reversed the inhibitory effect of amarogentin on the new synthesis of TNF-*α*.

### 3.2. Effect of Amarogentin on Histamine Induced IL-6 Production in Human Keratinocytes

**Figure 2:**
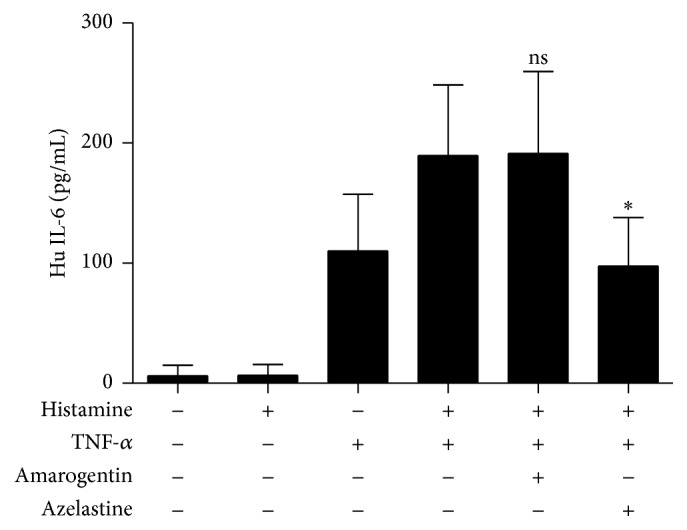
Impact of amarogentin on IL-6 secretion in stimulated HaCaT keratinocytes. HaCaT cells were costimulated with 100 *μ*M histamine and 25 ng/mL TNF-*α* and preincubated with amarogentin (100 *μ*M) or the control azelastine (24 *μ*M) for 30 minutes. After 24 h IL-6 expression was assessed in the cell supernatant. The data are shown with standard deviation (SEM) and were analyzed with the unpaired, two-tailed *t*-test (^*∗*^ *p* < 0.05; ns: not statistically significant; *n* = 3).

The human keratinocyte cell line HaCaT produces IL-6 after costimulation with TNF-*α* and histamine [5]. To test the effect of amarogentin in this setting, HaCaT cells were preincubated with 100 *μ*M amarogentin or 24 *μ*M azelastine for 30 min, before stimulation with 10^−5^ M histamine and/or 25 ng/mL TNF-*α* for 24 hours. The used concentrations of amarogentin and azelastine were not cytotoxic for HaCaT cells. In contrast to amarogentin, azelastine inhibited the IL-6 release from HaCaT cells (Figure 2).

### 3.3. Effect of Amarogentin on Histamine and TNF-*α* Induced IL-8 and MMP-1 Production in Human Keratinocytes

**Figure 3:**
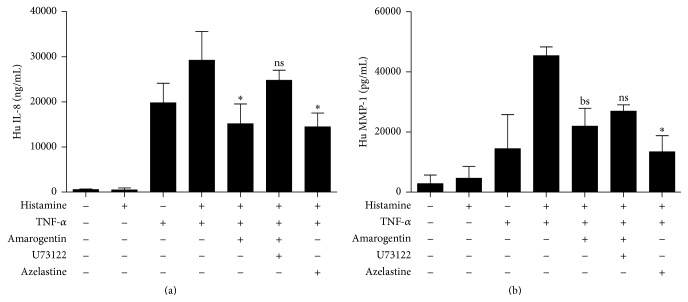
Amarogentin does not inhibit IL-8 and MMP-1 secretion in stimulated HaCaT keratinocytes. HaCaT cells were costimulated with 10 *μ*M histamine and 25 ng/mL TNF-*α* and preincubated with amarogentin (100 *μ*M) or azelastine (24 *μ*M) for 30 minutes. A part of the amarogentin stimulated group was preincubated for 30 min with the PLC inhibitor U73122 (10 *μ*M). After 24 h IL-8 (a) and MMP-1 (b) expression was assessed in the cell supernatant. The data are shown with standard deviation (SEM) and were analyzed with the unpaired, two-tailed *t*-test (^*∗*^ *p* < 0.05; bs: borderline significant 0.05 < *p* < 0.06, ns: not statistically significant; *n* = 3).

IL-8 is a chemoattractant for neutrophils and T cells and may initiate cutaneous inflammation. To assess if amarogentin inhibits IL-8 cytokine production, amarogentin treated HaCaT cells were stimulated with 10 *μ*M histamine. However, histamine stimulation alone did not induce IL-8 expression in HaCaT cells (Figure 3(a)); therefore, 25 ng/mL TNF-*α* was additionally applied to synergistically increase this secretion, because histamine and TNF-*α* are secreted together from mast cells. This effect could be inhibited by amarogentin as well as azelastine. To enable neutrophils and T cells the transmigration through the basement membrane, the expression of the matrix metalloproteinase MMP-1 is required. Again, histamine alone only marginally enhanced the production of MMP-1 (Figure 3(b)), whereas TNF-*α* increased synergistically this expression. Amarogentin as well as azelastine could inhibit the release of MMP-1 in histamine and TNF-*α* costimulated HaCaT cells. U73122, a PLC inhibitor, which can inhibit the bitter taste receptor signaling pathway, reversed the inhibitory effect of amarogentin on MMP-1 and IL-8 secretion in stimulated HaCaT cells. This effect was pronounced related to the IL-8 secretion. The MMP-1 release could only partly be restored.

Similarly, amarogentin as well as azelastine could inhibit the release of MMP-1 in histamine and IFN-*γ* (T cell derived cytokine) costimulated HaCaT cells (supplementary data 2).

### 3.4. Effect of Amarogentin on Histamine and TNF-*α* Induced NGF Expression in Human Keratinocytes

**Figure 4:**
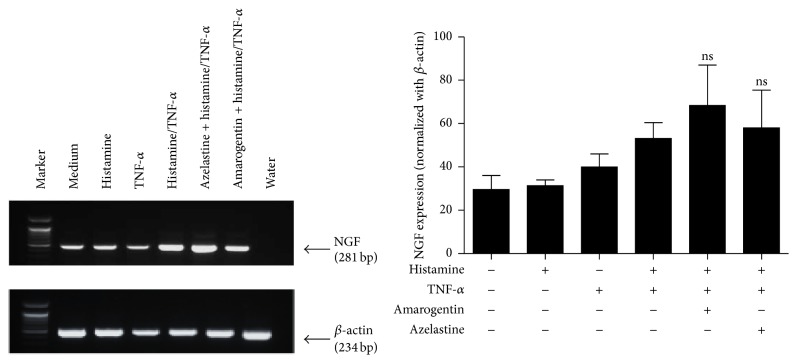
Amarogentin inhibits NGF secretion in stimulated HaCaT keratinocytes. HaCaT cells were costimulated with 10 *μ*M histamine and 25 ng/mL TNF-*α* and preincubated with amarogentin (100 *μ*M) or the control azelastine (24 *μ*M) for 30 minutes. After 4 h NGF RNA expression was assessed. The data are shown with standard deviation (SEM) and were analyzed with the unpaired, two-tailed *t*-test (ns: not statistically significant; *n* = 3).

Keratinocytes constitutively secrete low amounts of NGF; this secretion can be increased by histamine (10^−5^ M) and TNF-*α* (25 ng/mL) costimulation. Histamine alone only has a small effect [5]. As NGF expression is linked with inflammation and pruritus we analyzed if amarogentin could block the release of NGF. However, preincubation with neither amarogentin (100 *μ*M) nor azelastine (24 *μ*M) for 1 h could inhibit histamine and TNF-*α* induced NGF mRNA-expression after 4 h incubation (Figure 4).

## 4. Discussion

Keratinocytes are actively engaged in skin inflammatory responses by releasing proinflammatory cytokines (e.g., IL-6), chemokines (e.g., IL-8), or matrix metalloproteinases (e.g., MMP-9) [4, 5]. The migration of immune cells across the basal lamina is an important process during inflammation that is controlled by the composition of the basement membrane and the balance of proteases, cytokines, and chemokines. MMPs influence all these parameters and are highly expressed in inflammation of, for example, lesional acute eczema [17]. In human skin high amounts of histamine are secreted by activated mast cells or basophils. These histamine induced changes in keratinocytes lead to increased transmigration of T cells through an artificial basement membrane and indicate that the increased MMP expression is functional [4]. In our study we could block histamine and TNF-*α* induced MMP-1 release by amarogentin to a similar extent as with the histamine antagonist azelastine. It is suggested that keratinocytes enhance the effect of histamine due to elevated H1-receptor expression by TNF-*α* and amarogentin might influence this effect. Furthermore TAS2Rs are expressed and functional in keratinocytes [14] and activation of TAS2Rs might also occur in HaCaT cells and explain the effectiveness of the bitter compound amarogentin. Activated bitter taste receptors lead via the G-protein *α*-gustducin to the activation of phospholipase C-*β*2 (PLC*β*2) and the formation of inositol trisphosphate as well as triacylglycerol and eventually to the opening of the transient receptor potential cation channel 5 [18]. The effect of amarogentin in the HaCaT cells could be reversed by U73122, a PLC inhibitor that is a key enzyme of the bitter taste receptor signaling.

These data suggest that the inhibitory effect of amarogentin on SP-induced TNF-*α* release is mediated via bitter taste receptor signaling.

**Figure 5:**
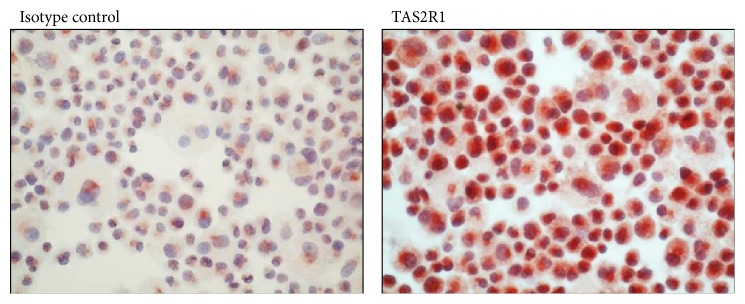
Immunocytochemical staining against TAS2R1. The human mast cell line LAD-2 was stained with an isotype control antibody or antibodies against TAS2R1.

In addition, TAS2Rs were upregulated in human leukocytes of patients suffering from severe inflammatory therapy-resistant asthma compared to healthy controls. Orsmark-Pietras and colleagues demonstrated that the TAS2R agonists' chloroquine and denatonium inhibit the release of several proinflammatory cytokines including TNF-*α*, IL-1*α*, and IFN-*γ* from blood leucocytes after LPS stimulation [19]. The inhibitory effect of chloroquine on the release of histamine from rat mast cells after stimulation with compound 48/80 or calcium ionophore A42187 was already described 25 years ago [20, 21] and can now also be related to bitter taste receptor expression. Very recently Ekoff and colleagues analyzed the expression of 9 TAS2Rs on human mast cells [22]. Furthermore, they studied the effect of 4 TAS2R agonists including chloroquine and denatonium on human mast cell-mediated release of histamine and prostaglandin D2. After activation via IgE-receptor cross-linkage both cord blood-derived mast cells and the mast cell line HMC1.2 expressed all tested TAS2Rs (TAS2R3, TAS2R4, TAS2R5, TAS2R10, TAS2R13, TAS2R14, TAS2R19, TAS2R20, and TAS2R46). Moreover, agonists known to bind to these particular TAS2Rs significantly inhibited the release of histamines from IgE-stimulated mast cells. This suggests that TAS2R agonists may have an anti-inflammatory action. However, the 4 different TAS2R agonists used in the study of Ekhoff and colleagues displayed varying capacities of inhibition and the mechanism behind this pattern is still unknown. As amarogentin can activate TAS2R1, TAS2R4, TAS2R39, TAS2R43, TAS2R46, TAS2R47, and TAS2R50, at least the TAS2R 4 and TAS2R46 overlap with the study from Ekhoff and colleagues and could be activated by amarogentin. In addition, we could demonstrate that the mast cell line LAD-2 expresses also TAS2R1 that can be activated by amarogentin (Figure 5). Furthermore, only TNF-*α* but not IL-6 or IFN-*γ* enhances the production of NGF in human keratinocytes via the Raf-1/MERK/ERK pathway in human keratinocytes and ERK-Inhibitors can inhibit this signaling pathway [11]. Such positive feedback loops of TNF-*α*/NGF may amplify* in vivo* skin inflammation as NGF induces neurons to synthesize substance P [23]. Although amarogentin does not inhibit the degranulation of LAD-2 cells and does not act as human mast cell stabilizer, it can inhibit the new synthesis of TNF-*α*. This effect could be reversed by the PLC inhibitor U73122 that was already described as substance to inhibit the bitter taste receptor pathway in rat neuronal cells [16]. The data suggest that the inhibitory effect of amarogentin on IL-8 and MMP-1 release is at least partly mediated via bitter taste receptor signaling.

These results supplement our previous observations that the treatment of HaCaT cells with amarogentin influences the differentiation process [14].

**Figure 6:**
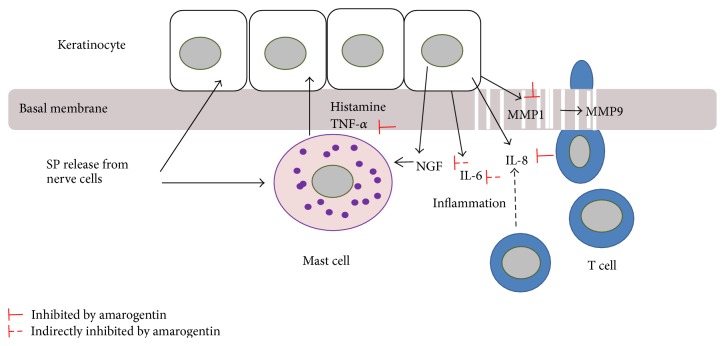
Simplified working hypothesis for the reaction mechanism of TAS2R agonists on cellular interactions among mast cells, T cells, and keratinocytes in the epidermis and uppermost dermis of inflamed skin. The reaction might be triggered by substance P (SP) released from nerve cells. The TAS2R agonist amarogentin inhibits the secretion of TNF-*α* from mast cells. Furthermore, the section of MMP-1 and IL-8 is directly inhibited by amarogentin in histamine and TNF-*α* costimulated human keratinocytes. Reduced MMP-1 expression results in reduced degradation of the basement membrane and reduced T cell transmigration to the epidermis. This may reduce the secretion of inflammatory cytokines by T cells in the epidermis.

A simplified working hypothesis for the action of amarogentin on mast cells, keratinocytes, and T cells is shown in Figure 6. First amarogentin reduces the expression of newly synthesized TNF-*α* in SP-stimulated mast cells. Second histamine and TNF-*α* induce IL-8 as well as MMP-1 release; these secretions could be blocked by amarogentin. Furthermore, amarogentin indirectly influences NGF and IL-6 expression by inhibiting the new synthesis of TNF-*α* in mast cells after stimulation with SP2.

Until now the involvement of TAS2Rs was not described in chronic inflammatory diseases such as atopic dermatitis and psoriasis. However, we could demonstrate that the expressions of TAS2Rs are downregulated in psoriasis (data not shown), so that a stimulation of the remaining receptors may influence the skin condition. The endogenous ligands for TAS2Rs in keratinocytes are still unknown, but it can be speculated that bitter tasting amino acids of the natural moisturizing factors of the skin (e.g., such as tyrosine or histidine) may act in this way.

## 5. Conclusion

The bitter compound amarogentin may modulate the milieu of inflamed skin. Mast cells, T cells, and keratinocytes are in close relationship with one another in the uppermost dermis during inflammation and mast cells and keratinocytes express TAS2Rs and react on bitter compounds. Therefore, TAS2R signaling might modulate skin inflammation in addition to already described functions of bitter taste receptors such as bronchodilation, hormone secretion, and bacterial killing. However, it must still be clarified if this effect actually takes place* in vivo* in pathologic processes such as psoriasis and eczema.

## Supplementary Material

Supplementary data 1: Cell viability of LAD-2 cells after amarogentin and azelastine treatment Amarogetin (100 µM) and azelastine (24 µM) were used in not cytotoxic concentrations. To test the cytotoxicity of the extracts LAD-2 cell viability was assessed with the ViaLight Plus ATP assay (Cambrex, Verviers, Belgium) according to the manufacturer's instruction. The method is based on the bioluminescent measurement of ATP that is present in metabolically active cells. Luciferase catalyzes the formation of light from ATP and luciferin. The emitted light intensity is directly proportional to the ATP concentration and is measured with a luminometer (Sirius HT, MWG).
Supplementary data 2: Effect of amarogentin on histamine and IFN-γ-induced MMP-1 production in human keratinocytes Histamine only slightly enhanced the production of MMP-1, whereas IFN-γ increased synergistically this expression. Amarogentin as well as azelastine could inhibit the release of MMP-1 in histamine and IFN-γ co-stimulated HaCaT cells.

## Abbreviations

NGF: Nerve growth factor
MMP: Matrix metalloproteinase
PLC: Phospholipase C
TNF-*α*: Tumor necrosis factor *α*
SP: Substance P
TAS2R: Bitter taste receptor.

## References

[1] Tiligada E. (2012). Editorial: is histamine the missing link in chronic inflammation?. *Journal of Leukocyte Biology*.

[2] Harvima I. T. (2008). Induction of matrix metalloproteinase-9 in keratinocytes by histamine. *Journal of Investigative Dermatology*.

[3] Shirasaki H., Kanaizumi E., Seki N., Himi T. (2012). Localization and upregulation of the nasal histamine H1 receptor in perennial allergic rhinitis. *Mediators of Inflammation*.

[4] Gschwandtner M., Purwar R., Wittmann M. (2008). Histamine upregulates keratinocyte MMP-9 production via the histamine H1 receptor. *Journal of Investigative Dermatology*.

[5] Kohda F., Koga T., Uchi H., Urabe K., Furue M. (2002). Histamine-induced IL-6 and IL-8 production are differentially modulated by IFN-gamma and IL-4 in human keratinocytes. *Journal of Dermatological Science*.

[6] Kanda N., Watanabe S. (2003). Histamine enhances the production of nerve growth factor in human keratinocytes. *Journal of Investigative Dermatology*.

[7] Giustizieri M. L., Albanesi C., Fluhr J., Gisondi P., Norgauer J., Girolomoni G. (2004). H1 histamine receptor mediates inflammatory responses in human keratinocytes. *Journal of Allergy and Clinical Immunology*.

[8] Han Y.-P., Tuan T.-L., Hughes M., Wu H., Garner W. L. (2001). Transforming growth factor-*β* - and tumor necrosis factor-*α* -mediated induction and proteolytic activation of MMP-9 in human skin. *Journal of Biological Chemistry*.

[9] Tsoureli-Nikita E., Watson R. E. B., Griffiths C. E. M. (2006). Photoageing: the darker side of the sun. *Photochemical and Photobiological Sciences*.

[10] Roggenkamp D., Köpnick S., Stäb F., Wenck H., Schmelz M., Neufang G. (2013). Epidermal nerve fibers modulate keratinocyte growth via neuropeptide signaling in an innervated skin model. *Journal of Investigative Dermatology*.

[11] Takaoka K., Shirai Y., Saito N. (2009). Inflammatory cytokine tumor necrosis factor-alpha enhances nerve growth factor production in human keratinocytes, HaCaT cells. *Journal of Pharmacological Sciences*.

[12] Kumar I. V. M. L. R. S., Paul B. N., Asthana R., Saxena A., Mehrotra S., Rajan G. (2003). Swertia chirayita mediated modulation of interleukin-1beta, interleukin-6, interleukin-10, interferon-gamma, and tumor necrosis factor-alpha in arthritic mice. *Immunopharmacology and Immunotoxicology*.

[13] Meyerhof W., Batram C., Kuhn C. (2010). The molecular receptive ranges of human TAS2R bitter taste receptors. *Chemical Senses*.

[14] Wölfle U., Elsholz F. A., Kersten A., Haarhaus B., Müller W. E., Schempp C. M. (2015). Expression and functional activity of the bitter taste receptors TAS2R1 and TAS2R38 in human keratinocytes. *Skin Pharmacology and Physiology*.

[15] Kempuraj D., Huang M., Kandere-Grzybowska K. (2003). Azelastine inhibits secretion of IL-6, TNF-alpha and IL-8 as well as NF-kappaB activation and intracellular calcium ion levels in normal human mast cells. *International Archives of Allergy and Immunology*.

[16] Akiyoshi T., Tanaka N., Nakamura T., Matzno S., Shinozuka K., Uchida T. (2007). Effects of quinine on the intracellular calcium level and membrane potential of PC 12 cultures. *Journal of Pharmacy and Pharmacology*.

[17] Purwar R., Kraus M., Werfel T., Wittmann M. (2008). Modulation of keratinocyte-derived MMP-9 by IL-13: a possible role for the pathogenesis of epidermal inflammation. *Journal of Investigative Dermatology*.

[18] Amrein H., Bray S. (2003). Bitter-sweet solution in taste transduction. *Cell*.

[19] Orsmark-Pietras C., James A., Konradsen J. R. (2013). Transcriptome analysis reveals upregulation of bitter taste receptors in severe asthmatics. *European Respiratory Journal*.

[20] Green K. B., Lim H. W. (1989). Effects of chloroquine on release of mediators from mast cells. *Skin Pharmacology*.

[21] Nosal R., Drabikova K., Pecivova J. (1991). Effect of chloroquine on isolated mast cells. *Agents Actions*.

[22] Ekoff M., Choi J.-H., James A., Dahlén B., Nilsson G., Dahlén S.-E. (2014). Bitter taste receptor (TAS2R) agonists inhibit IgE-dependent mast cell activation. *Journal of Allergy and Clinical Immunology*.

[23] Lindsay R. M., Harmar A. J. (1989). Nerve growth factor regulates expression of neuropeptide genes in adult sensory neurons. *Nature*.

