# Supplementary material for: Amarogentin Displays Immunomodulatory Effects in Human Mast Cells and Keratinocytes
Source: Mediators Inflamm. 2015 Oct 27;2015:630128. doi: 10.1155/2015/630128 (PMC4639662; doi:10.1155/2015/630128)
Supplement: Supplementary file 1 — Supplementary data 1: Cell viability of LAD-2 cells after amarogentin and azelastine treatment Amarogetin (100 µM) and azelastine (24 µM) were used in not cytotoxic concentrations. To test the cytotoxicity of the extracts LAD-2 cell viability was assessed with the ViaLight Plus ATP assay (Cambrex, Verviers, Belgium) according to the manufacturer's instruction. The method is based on the bioluminescent measurement of ATP that is present in metabolically active cells. Luciferase catalyzes the formation of light from ATP and luciferin. The emitted light intensity is directly proportional to the ATP concentration and is measured with a luminometer (Sirius HT, MWG). Supplementary data 2: Effect of amarogentin on histamine and IFN-γ-induced MMP-1 production in human keratinocytes Histamine only slightly enhanced the production of MMP-1, whereas IFN-γ increased synergistically this expression. Amarogentin as well as azelastine could inhibit the release of MMP-1 in histamine and IFN-γ co-stimulated HaCaT cells. [file 630128.f1.pptx]

## Slide 1
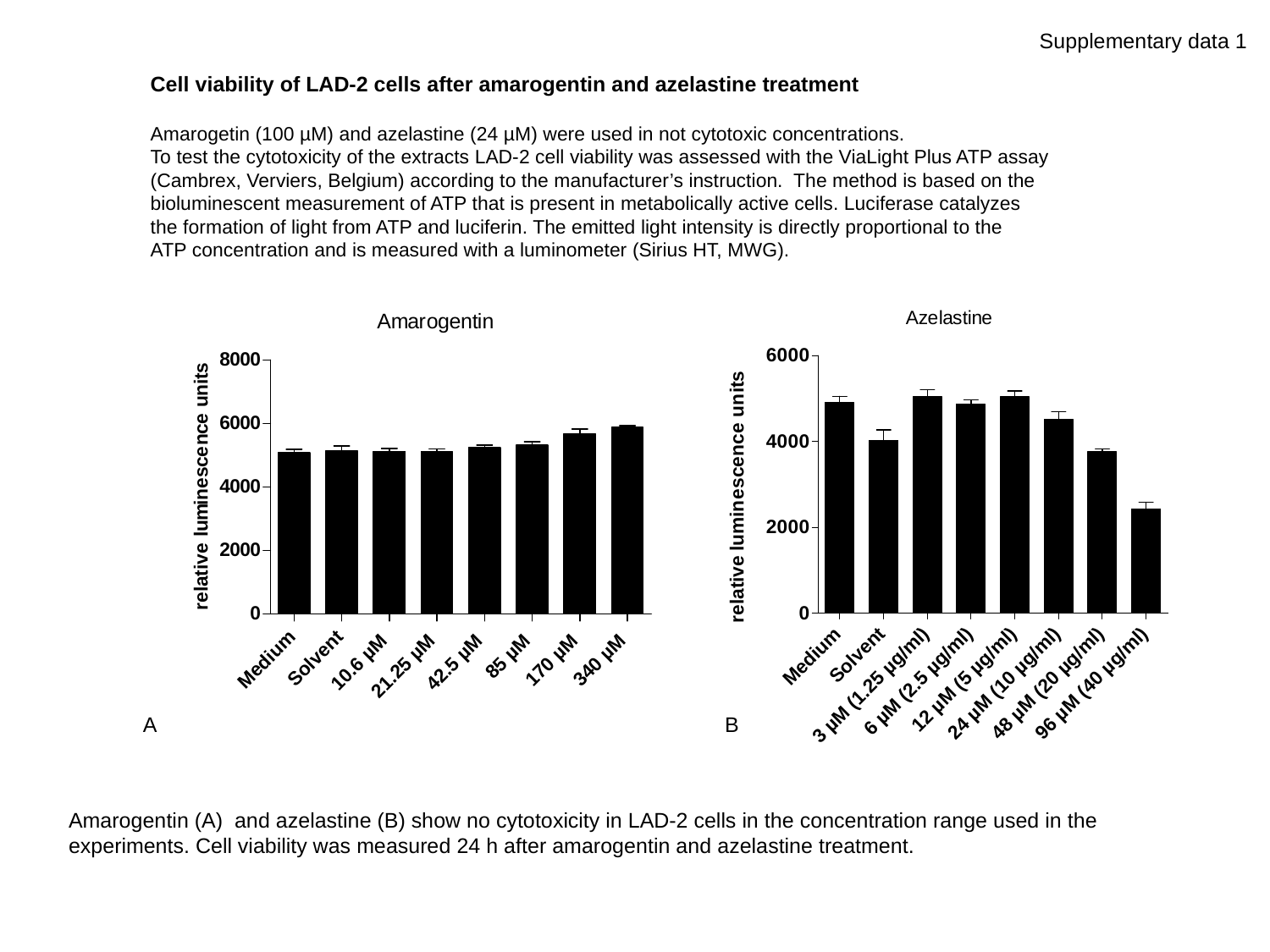

Supplementary data 1
Cell viability of LAD-2 cells after amarogentin and azelastine treatment
Amarogetin (100 µM) and azelastine (24 µM) were used in not cytotoxic concentrations.
To test the cytotoxicity of the extracts LAD-2 cell viability was assessed with the ViaLight Plus ATP assay
(Cambrex, Verviers, Belgium) according to the manufacturer’s instruction. The method is based on the
bioluminescent measurement of ATP that is present in metabolically active cells. Luciferase catalyzes
the formation of light from ATP and luciferin. The emitted light intensity is directly proportional to the
ATP concentration and is measured with a luminometer (Sirius HT, MWG).
A
B
Amarogentin (A) and azelastine (B) show no cytotoxicity in LAD-2 cells in the concentration range used in the experiments. Cell viability was measured 24 h after amarogentin and azelastine treatment.
