# Supplementary material for: Amarogentin Displays Immunomodulatory Effects in Human Mast Cells and Keratinocytes
Source: Mediators Inflamm. 2015 Oct 27;2015:630128. doi: 10.1155/2015/630128 (PMC4639662; doi:10.1155/2015/630128)
Supplement: Supplementary file 2 [file 630128.f2.pptx]

## Slide 1
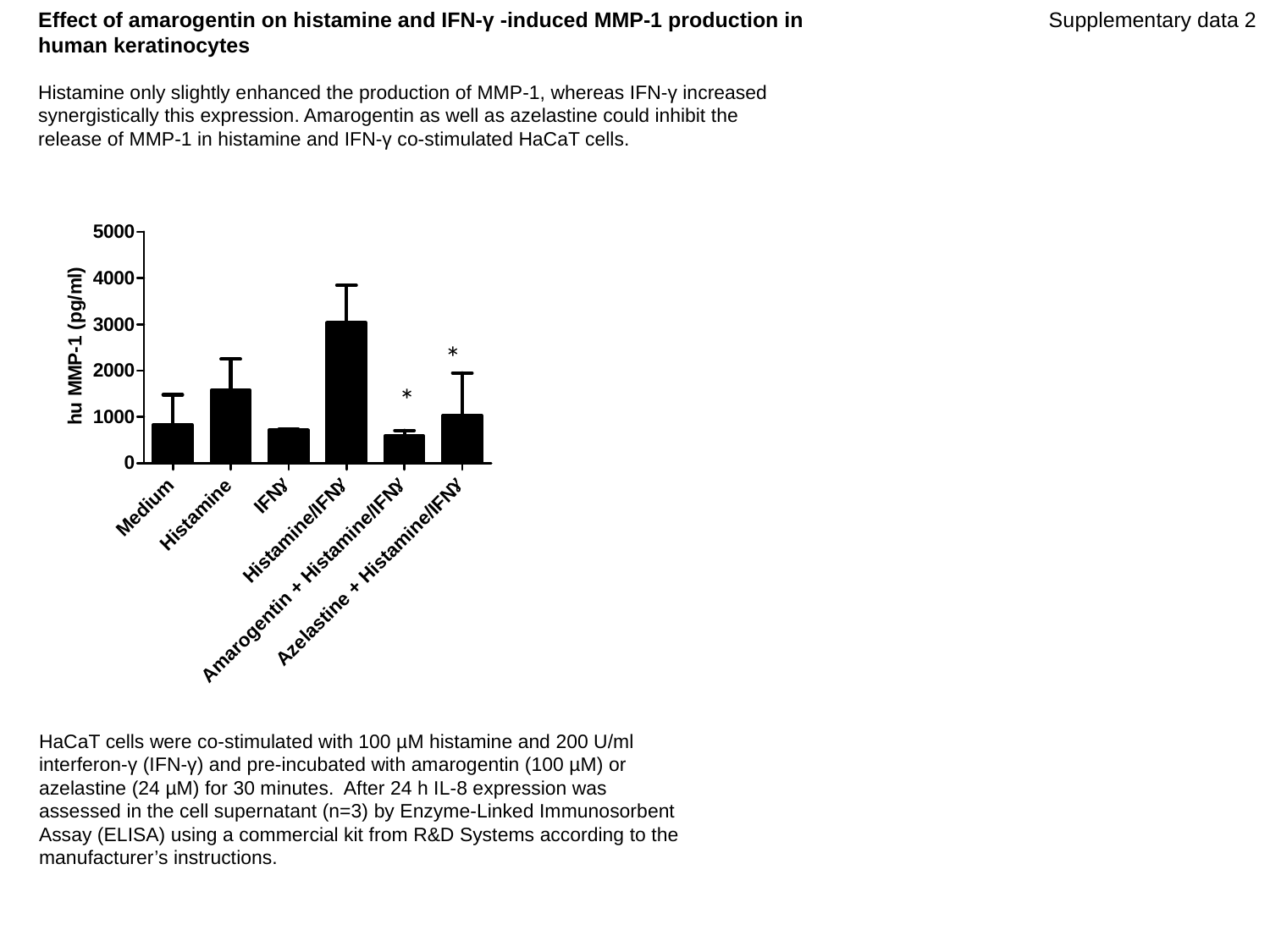

Effect of amarogentin on histamine and IFN-γ -induced MMP-1 production in
human keratinocytes
Histamine only slightly enhanced the production of MMP-1, whereas IFN-γ increased
synergistically this expression. Amarogentin as well as azelastine could inhibit the
release of MMP-1 in histamine and IFN-γ co-stimulated HaCaT cells.
Supplementary data 2
*
*
HaCaT cells were co-stimulated with 100 µM histamine and 200 U/ml
interferon-γ (IFN-γ) and pre-incubated with amarogentin (100 µM) or
azelastine (24 µM) for 30 minutes. After 24 h IL-8 expression was
assessed in the cell supernatant (n=3) by Enzyme-Linked Immunosorbent
Assay (ELISA) using a commercial kit from R&D Systems according to the
manufacturer’s instructions.
